# Development of Ketoprofen Impurity A (1-(3-Benzoylphenyl)ethanone) as a Certified Reference Material for Pharmaceutical Quality Control

**DOI:** 10.3390/ph18010059

**Published:** 2025-01-07

**Authors:** Nikolay A. Shulga, Vladimir I. Gegechkori, Natalya V. Gorpinchenko, Valery V. Smirnov, Sergey P. Dementyev, Galina V. Ramenskaya

**Affiliations:** Department of Pharmaceutical and Toxicological Chemistry named after Arzamastsev, Institute of Pharmacy, Federal State Autonomous Educational Institution of Higher Education I.M. Sechenov First Moscow State Medical University of the Ministry of Health of the Russian Federation (Sechenov University), 8-2 Trubetskaya Str., 119991 Moscow, Russia

**Keywords:** related substances, ketoprofen impurity, reference material, purification

## Abstract

**Background/Objectives**: Reference materials are essential for ensuring the accuracy and traceability of measurements in the quality control of medicinal products. This study explores new principles for the preparation of impure materials of active pharmaceutical substances, focusing on 1-(3-benzoylphenyl)ethanone ketoprofen impurity A (*European Pharmacopoeia*) as the reference material. **Methods**: The reference material was synthesised from commercially available acetanilide and benzoyl chloride. The obtained product was purified using preparative chromatography and characterised by infrared spectroscopy (IR), 1H and 13C nuclear magnetic resonance (NMR), and mass spectrometry. The structure was verified using primary research methods to confirm its identity as the target product. **Results**: The characterisation confirmed the structure and purity of 1-(3-benzoylphenyl)ethanone, achieving a purity of 99.86%, meeting regulatory documentation requirements. The synthesised product was demonstrated to be identical to the target compound and suitable for use as a reference material. **Conclusions**: The developed method provides a robust approach for the preparation and characterisation of 1-(3-benzoylphenyl)ethanone, enabling its use as a certified reference material in the quality control of medicinal products. This approach ensures compliance with regulatory standards and enhances the reliability of pharmaceutical quality assurance practices.

## 1. Introduction

Related impurity testing is a necessary and important part of the quality control of medicinal products (MPs) and active pharmaceutical ingredients (APIs). The purity of a drug impacts its safety and efficacy; however, in most cases, it is either impossible or impractical to obtain drugs in an absolutely pure form due to the significant increase in the cost of production [1,2].

Many documents regulate the standardisation of impurities in drugs and APIs, including the International Council for Harmonisation guidelines (in particular, Q3A and Q3B). The impurity definitions in these documents are similar and are as follows: “Any component of the new drug substance that is not the chemical entity defined as the impurity” [3,4].

After establishing the limits of impurity content in monographs, specifications, and studies on the indicator “related impurities”, the limits of impurity content are widely used for the routine quality control of drugs and APIs in pharmaceutical production contexts, as well as for other purposes. Generally, such studies are carried out using chromatographic or spectroscopic methods [5,6]

In this context, the use of reference materials (RMs) is an indispensable part of the quality control of medicinal products and APIs in relation to the indicator “related impurities”. This is due to the fact that these RMs ensure the metrological traceability and accuracy of the measurement results. It is important to note that the use of RMs not only ensures the comparability of the results of the analyses between different laboratories but also guarantees that the obtained data comply with the requirements of various specifications. Thus, the use of RMs plays a key role in ensuring the reliability and validity of analytical studies with regard to determining the contents of related impurities [7,8]

The raw materials required for the identification and quantification of APIs are most often a part of the batch of APIs used for the production of the MP. Such raw materials are characterised by high commercial availability, low cost, and wide supply on the market; this allows the purest option to be chosen, which, therefore, is suitable for RM creation. In the case of RMs used for the control of related impurities in APIs, the source material for certification as an RM is usually unavailable. RMs of impurities can be pure substances of the respective impurities, mixtures of these impurities, as well as APIs enriched with the given impurity. The commercial availability of such materials is low, and their cost can be higher than that of the APIs [8,9]. In light of this, it is urgent to create and develop new approaches to obtain materials that are suitable for certification as RMs.

Ketoprofen is one of the most frequently prescribed non-steroidal anti-inflammatory drugs used for the treatment of pain and inflammatory processes of moderate to mild severity. The conditions for which it is prescribed include arthritis of various origins, post-traumatic and post-operative pain syndrome, and myalgia and neuralgia of various geneses, as well as some other conditions [10]

**Figure 1:**
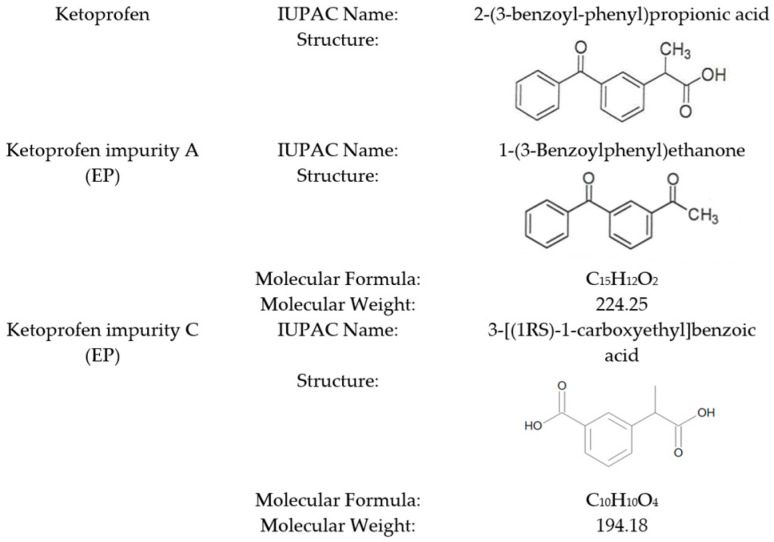
Chemical characteristics of ketoprofen impurities (A and C).

According to the *European Pharmacopoeia*, ketoprofen has six specified impurities, and the calculation of the quantitative impurity content using RMs is applied only for two of them: impurity A and impurity C (Figure 1) [11,12]

Structure–activity relationships are known to lead to a risk of activity or toxicity of impurities in MPs [13]. For example, some studies indicate the phototoxicity of ketoprofen oxidation products, which include ketoprofen impurity A [14,15]. Its toxicity is one of the reasons that RMs are used in pharmaceutical quality control of ketoprofen for the quantification of the impurity. Therefore, it is necessary to develop new methods for obtaining and characterising this impurity.

The aim of this study is to develop an effective and reproducible method for synthesising and characterising high-purity 1-(3-Benzoylphenyl)ethan-1-one (ketoprofen impurity A) for use as an RM. The material’s authenticity and structure were validated using IR, ^1^H and ^13^C NMR spectroscopy, and mass spectrometry, ensuring compliance with regulatory standards and analytical accuracy. The combined use of these three independent methods allows the structure and authenticity of the compound to be accurately established [11,16,17,18].

## 2. Results and Discussion

### 2.1. Selection of the Method for Obtaining the Material

The following characteristics of the production method are important when selecting the method used to obtain an API impurity material for use as an RM:

- Availability of raw materials;
- Economic efficiency;
- The smallest number of stages of production;
- The highest purity of the RM material.

Ketoprofen impurity A is one of the intermediates of ketoprofen synthesis, where 1-(3-benzoylphenyl)ethenone reacts with ethyl chloroacetate, followed by hydrolysis and decarboxylation, to yield ketoprofen. This mechanism can be considered as one of the possible pathways of impurity formation in the final ketoprofen product [19,20,21].

Another mechanism for the formation of 1-(3-benzoylphenyl)ethenone may be the degradation of ketoprofen as a result of an oxidative process. Thus, in their 2014 publication, Feng et al. indicated the possibility of oxidative degradation of ketoprofen affecting 1-(3-benzoylphenyl)ethenone. The same result was obtained by Illes et al. in 2014 [22,23].

As mentioned above, the impurity materials used as RMs can be obtained using various methods, as well as combinations thereof. Among such methods, the following can be highlighted:

- Organic synthesis;
- Enrichment of APIs or other products with an appropriate impurity;
- Separation of the impurity from the API or other product.

Therefore, based on the pathways of impurity formation in the ketoprofen API, we can assume two ways in which 1-(3-benzoylphenyl)ethenone material can be obtained: through organic synthesis or enrichment of the ketoprofen API with impurity A via degradation. Oxidative processes in APIs are not selective and are usually difficult to control, and the final product will require additional purification steps due to the large number of byproducts [24]. Therefore, we chose the path of organic synthesis due to the absence of these disadvantages.

**Figure 2:**
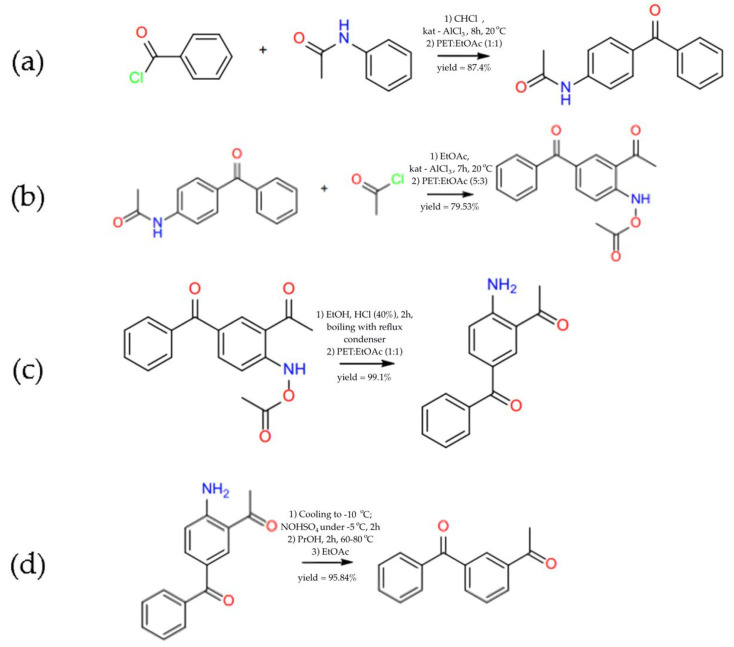
Scheme of material preparation for 1-(3-benzoylphenyl)ethenone: (**a**) preparation of 4-acetamidobenzophenone; (**b**) preparation of 2-acetamido-5-benzoyl-acetophenone; (**c**) preparation of 2-acetyl-4-benzoylaniline; (**d**) preparation of 1-(3-benzoylphenyl)ethenone.

The material 1-(3-benzoylphenyl)ethenone was prepared according to the following reaction scheme (shown in Figure 2).

**Figure 3:**
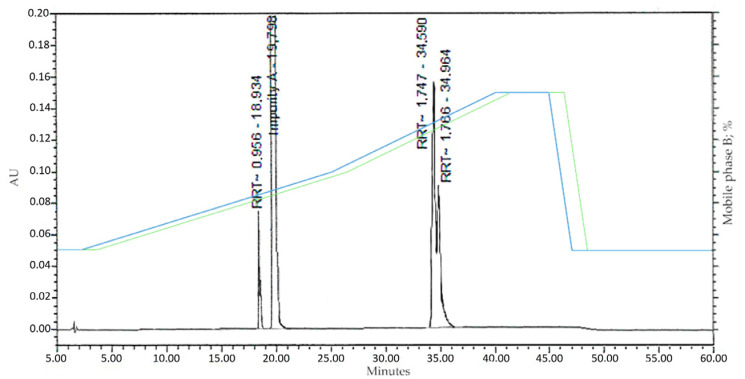
Chromatogram of crude material 1-(3-benzoylphenyl)ethenone. Blue line: programme gradient; green line: delayed elution gradient.

The obtained material was a light-yellow crystalline powder. A total of 20.3 g of 1-(3-benzoylphenyl)ethenone was obtained for further purification and characterisation for use as RM. The purity of the material, determined via analytical high-performance liquid chromatography (HPLC), was 90.45% (internal normalisation method). The data are presented in Figure 3 and Table 1.

### 2.2. Purification

The purity of a RM is one of the key characteristics reflecting the suitability of the material for its intended use. Thus, the *European Pharmacopoeia* states that, “Where a CRS is used to determine the content of a given impurity, the preferred minimum content is 95.0 percent; where this is achieved, the assigned content of the CRS is not given, and it is considered to be 100.0 percent” [11].

Preparative chromatography is one of the popular purification methods for the production of high-purity RMs. Preparative chromatography plays a key role in the production of high-purity compounds, including API impurities. Optimisation of the method ensures rapid purification of significant amounts of compounds and reduces the consumption and waste of solvents [25,26]. Therefore, we developed a method for the preparative purification of 1-(3-benzoylphenyl)ethenone.

The development and optimisation of the method is based on data obtained via analytical HPLC. Data on the retention time of the target compound and its impurities, as well as the composition of the mobile phase, were used. This included consideration of the gradient elution delay time, which reflects the time required for the mobile phase to reach the detector [27]. As a result of analysing the analytical HPLC data, the data shown in Table 2 were obtained.

**Figure 4:**
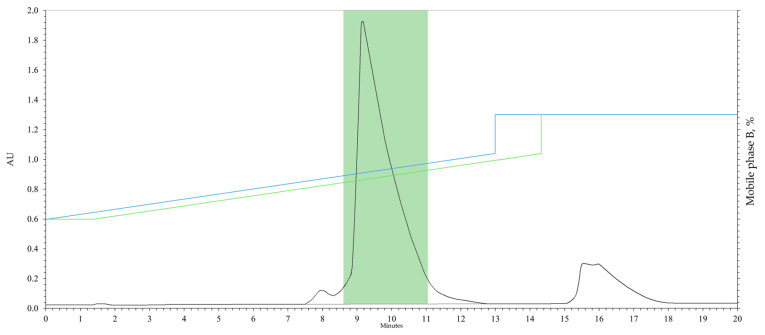
Chromatogram of preparative separation of the crude material 1-(3-benzoylphenyl)ethenone. Blue line: programme gradient; green line: delayed elution gradient; green zone: collection window of the target component fraction.

The developed preparative separation methodology utilises a flat gradient in the composition region of 42% mobile phase B to separate Impurity A and the compound with an RTT of 0.956. The gradient window was calculated as 42 ± 10%. Purification of compounds with RTTs of 1.747 and 1.766 is achieved using a step gradient to 65% mobile phase B. Figure 4 shows the preparative separation chromatogram of the crude material, 1-(3-benzoylphenyl)ethenone.

For purification and characterisation of the RM, 0.250 g of crude material was used. A solution of the crude material in methanol had a concentration of 0.0083 g/mL. In the experiment, 24 mL of this solution was used in 20 purification cycles, corresponding to 0.199 g of crude material. The fraction collection window was in the time interval 8.6–11.0 min.

The combined fractions were evaporated on a rotary vacuum evaporator at 40 °C and lyophilised. A total of 0.179 g of material was obtained after 20 purification cycles.

**Figure 5:**
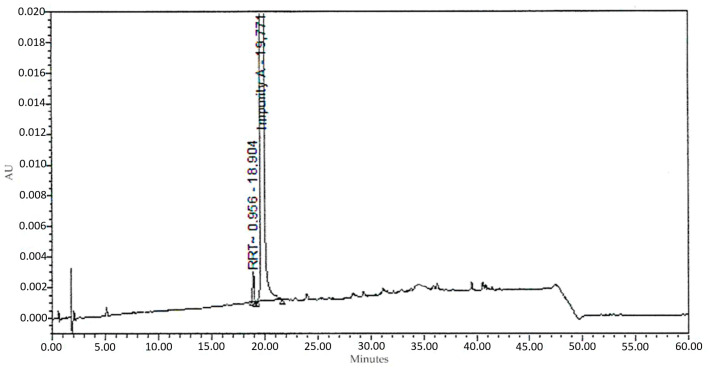
Chromatogram of the purified material 1-(3-benzoylphenyl)ethenone.

Repeated analysis with analytical HPLC revealed the purity of the crude material to be 99.86%, calculated via normalisation. The analytical HPLC data are presented in Figure 5 and Table 3.

After that, the obtained material was examined to confirm its structure. The material was analysed using IR, ^1^H and ^13^C NMR, and mass spectrometry techniques.

### 2.3. IR

The IR spectra (KBr), cm^−1^, were as follows: 691, 723, 820, 920, 918, 978, 1144, 1175, 1233, 1233, 1285, 1304, 1304, 1356, 1427, 1595, 1655, 1684, 2918, 3073.

**Figure 6:**
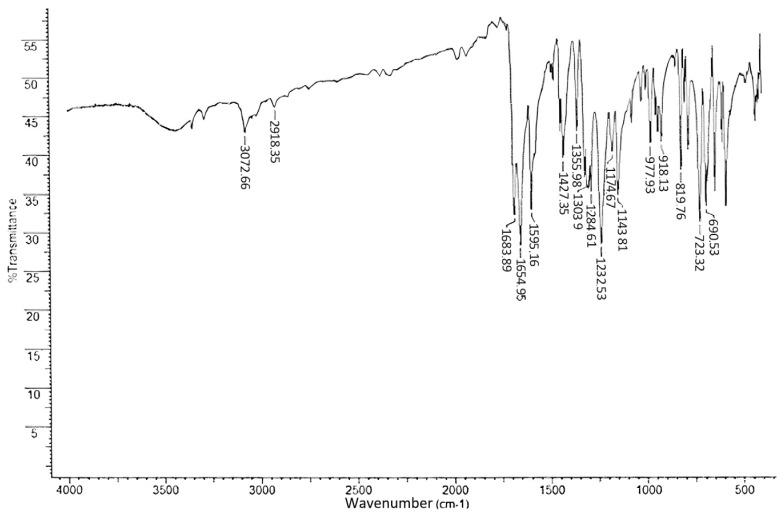
IR spectra of ketoprofen impurity A material.

The frequencies of the main absorption bands are provided in Table 4, and the IR spectrum is shown in Figure 6.

The bands in the region 3073–2918 cm^−1^ correspond to the valence vibrations of C–H bonds in methylene groups and aromatic ring methylene bonds. In the 1684 and 1655 cm^−1^ regions, two bands of valence vibrations for the C=O bonds of two carbonyl groups were observed, with the acetyl band presumably appearing in the higher-frequency (short-wavelength) region. Strain vibrations of the C–C bonds in aromatic fragments were observed in the region of 1595 cm^−1^. A band of antisymmetric deformation vibrations of the methylene group was observed in the 1427 cm^−1^ region, while the corresponding symmetric vibrations appeared as a band at 1356 cm^−1^. The valence antisymmetric and symmetric vibrations of the C–C(=O)–C group bonds appeared in the 1304–1233 cm^−1^ and 1175–1144 cm^−1^ intervals, respectively. The out-of-plane deformation vibrations of C–H bonds appeared as bands of average intensity in the region of 820 cm^−1^. Absorption bands of aromatic ring bonds in the region 723–691 cm^−1^ are due to deformation vibrations of C–C bonds.

Thus, the positions of the main absorption bands in the obtained IR spectrum do not contradict the chemical structure of 1-(3-benzoylphenyl)ethan-1-one.

### 2.4. ^1^H NMR Spectroscopy

**Figure 7:**
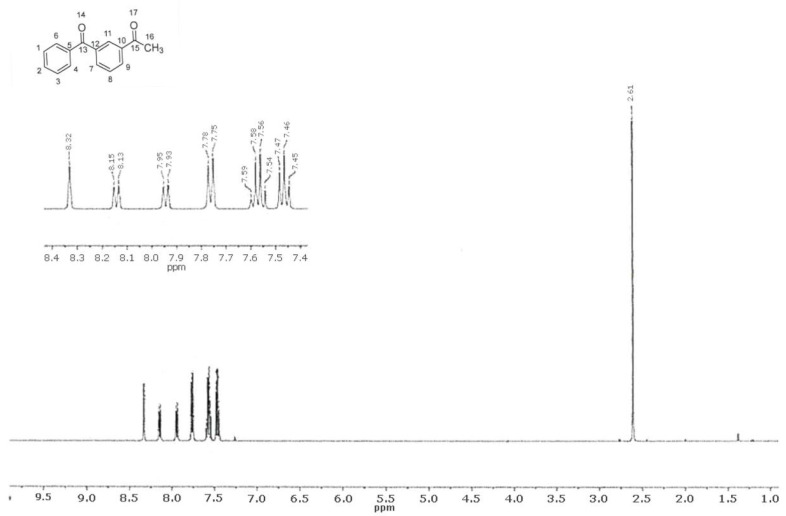
^1^H NMR spectra of ketoprofen impurity A material.

In the ^1^H NMR spectrum of the material, the proton signal of the methyl group C16 at 2.61 ppm was observed in the form of a singlet with a relative integral intensity equal to three protons. The aromatic protons at C1, C2, C3, and C8 were observed in triplet and quartet groups at 7.46 ppm and 7.57 ppm, respectively, with relative integral intensities of two protons each. The doublet observed in the 7.76 ppm region with spin–spin coupling (SSC) J = 7.3 Hz and an integrated intensity equal to two protons belongs to protons at C4 and C6. The two protons at C7 and C9 were observed as two doublets, one at 7.94 ppm with SSC J = 7.7 Hz and the other at 8.14 ppm with SSC J = 7.8 Hz. The singlet observed in the 8.33 ppm region refers to the proton at C11. The description and attribution of the signals are presented in Table 5, and the ^1^H NMR spectrum is presented in Figure 7.

### 2.5. ^13^C NMR Spectroscopy

**Figure 8:**
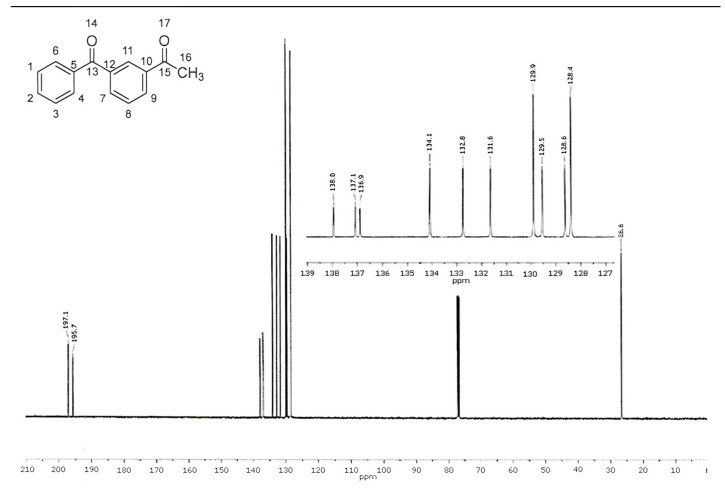
^13^C NMR spectra of 1-(3-benzoylphenyl)ethenone.

In the ^13^C NMR spectrum, the number of signals corresponds to the number of magnetically non-equivalent carbon atoms in the molecule. The position and multiplicity of signals in the ^13^C NMR spectra of the material align with the chemical structure of 1-(3-benzoylphenyl)ethan-1-one. The descriptions and attributions of the ^13^C NMR signals are presented in Table 6, and the ^13^C NMR spectrum is presented in Figure 8.

### 2.6. Mass Spectrometry

**Figure 9:**
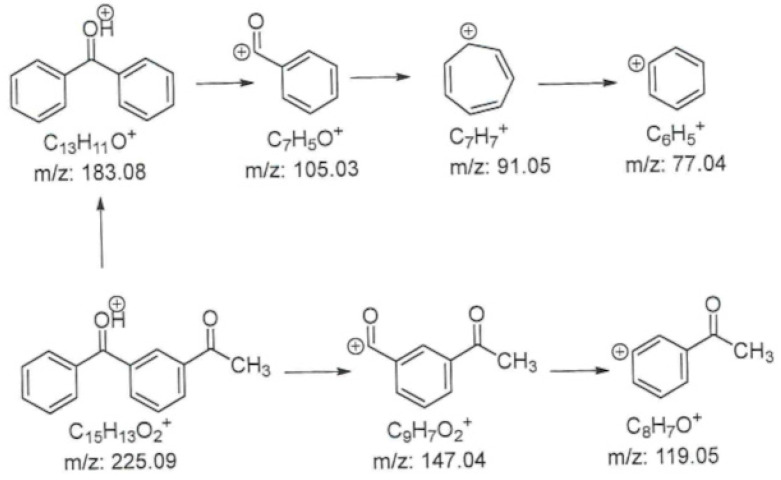
Probable fragmentation scheme of 1-(3-benzoylphenyl)ethenone.

The mass spectrum of the material showed a main peak at *m*/*z* [C_15_H_13_O_2_]^+^ = 225, which corresponds to the calculated value for the protonated form of ketoprofen impurity A, *m*/*z* [M + H]^+^. The tandem mass spectrum showed peaks at *m*/*z* [M + H]^+^ = 147 (C_9_H_7_O_2_^+^), *m*/*z* [M + H]^+^ = 119 (C_5_H_7_O^+^), *m*/*z* [M + H]^+^ = 105 (C_7_H_5_O^+^), *m*/*z* [M + H]^+^ = 91 (C_7_H_7_^+^), and *m*/*z* [M + H]^+^ = 77 (C_6_H_5_^+^). The probable fragmentation scheme is presented in Figure 9.

Thus, the authenticity of the material 1-(3-benzoylphenyl)ethanone (ketoprofen impurity A) was confirmed using primary research methods.

Confirmation of the structure of the material is the first step in establishing an RM. Further investigation of the material should focus on the quantification of the basic substance content and the assignment of content.

The mass balance method is a commonly used method in the practice of RM development, which involves determining the water content, residual organic solvents, and organic and inorganic impurities. The determination of water and inorganic impurities is carried out by recognised methods such as the Karl Fischer method and sulphate ash determination, respectively. Gas chromatography is the most popular method for investigating residual solvents. Information about the synthesis method and purification process can predict the presence of solvents in the materials that were used in these stages. For the control of organic impurities, we propose using the developed analytical HPLC technique.

## 3. Materials and Methods

### 3.1. Synthesis of 1-(3-Benzoylphenyl)ethanone

The following reagents were used in the synthesis of the crude material: benzoyl chloride (Acros Organics, purity ≥ 99%, Geel, Belgium); acetanilide (Sigma-Aldrich, purity ≥ 98%, St. Louis, MO, USA); aluminium chloride (Merck, purity ≥ 98%, Darmstadt, Germany); chloroform (Fisher Scientific, purity ≥ 99%, Loughborough Leicestershire, UK); hydrochloric acid, concentrated (Sigma-Aldrich, purity 36%, USA); sodium sulphate, anhydrous (Acros Organics, purity ≥ 99%, Belgium); petroleum ether (VWR Chemicals, purity ≥ 98%, Briare, France); ethyl acetate (Honeywell, purity ≥ 99.5%, Charlotte, NC, USA); acetyl chloride (Merck, purity ≥ 98%, Germany); ethanol (Sigma-Aldrich, purity ≥ 99.8%, USA); sodium carbonate (Fisher Scientific, purity ≥ 99%, UK); nitrosyl sulphate (Alfa Aesar, purity ≥ 97%, Haverhill, MA, USA); and propanol (Merck, purity ≥ 99.5%, Germany).

The synthesis involved 4 steps:

- Reagents: 16.4 g of benzoyl chloride, 15.8 g of acetanilide, 17.1 g of aluminium chloride. Reaction conditions: chloroform (400 mL), 8 h at room temperature, addition to ice water with HCl (8.3%), drying over Na_2_SO_4_, evaporation. Recrystallisation from a mixture of petroleum ether–ethyl acetate (1:1). The product of this stage is 24.4 g 4-Acetamidobenzophenone.
- Reagents: 24.4 g of 4-acetamidobenzophenone and 24.4 g of aluminium chloride. Reaction conditions: ethyl acetate (100 mL), slow addition of acetyl chloride (10 mL) over 20 min, reaction at room temperature for 7 h. Reaction is ended by adding ice water with HCl (8.3%), drying over Na_2_SO_4_ and evaporating over a vacuum. Recrystallisation from petroleum ether–ethyl acetate (5:3). The product of this stage is 22.8 g 2-Acetamido-5-benzoyl-acetophenone.
- Reagents: 22.8 g of 2-acetamido-5-benzoyl-yl-acetophenone. Reaction conditions: ethanol (50 mL), HCl 40% (20 mL), boiling for 2 h, addition of Na_2_CO_3_ solution with cooling, drying, and evaporation under a vacuum. Recrystallisation from petroleum ether–ethyl acetate (1:1). The product of this stage is 22.6 g 2-Acetyl-4-benzoylaniline.
- Reagents: 22.6 g of 2-acetamido-5-benzo-yl-acetophenone. Reaction conditions: cooling to −10 °C, addition of 50 mL of nitrosyl sulphate solution (34 g/100 mL) at −5 °C, stirring for 2 h, addition of propanol (50 mL), heating to 60–80 °C, reaction for 2 h. Extraction with ethyl acetate, drying, and evaporation under vacuum. The product of the stage is 20.3 g 1-(3-benzoylphenyl)ethenone.

### 3.2. Analytical HPLC of 1-(3-Benzoylphenyl)ethanone

The following solvents were used for the analytical HPLC of 1-(3-benzoylphenyl)ethenone: formic acid (≥99%, Sigma-Aldrich, USA) and acetonitrile (≥99.9%, Honeywell, USA).

This study was carried out under the following conditions: mobile phase (MP) A—deionised water/formic acid 0.1%; MP B—acetonitrile (ACN)/formic acid 0.1%; sample preparation—20 mg of material dissolved in 100 mL of MP A/MP B (50/50) mixture and filtered through 0.45 polyvinylidene fluoride (PVDF) filter; column thermostat set at 35 °C; autosampler thermostat set at 15 °C; flow rate—0.9 mL/min; injection volume—10 µL; diode array detector set at 233 nm; column—Zorbax Eclipse Plus C18 4.6 mm × 150 mm, 3.5 µm (Agilent Technologies, Santa Clara, CA, USA); HPLC system—Agilent Infinity 1290 LC (Agilent Technologies, USA). Gradient elution: 25% mobile phase B (0–2 min), linear increase to 50% (2–25 min), then to 75% (25–40 min); held until 45 min, returned to 25% (47 min), and maintained until the end of the run (60 min).

### 3.3. Preparative HPLC of 1-(3-Benzoylphenyl)ethanone

The following reagents and solvents were used in the preparative chromatography, according to the developed methodology: methanol (≥99.9%, Sigma-Aldrich, USA); formic acid (≥99%, Sigma-Aldrich, USA); and ACN (≥99.9%, Honeywell, USA).

Purification of 1-(3-benzoylphenyl)ethenone material was carried out under the following conditions: MP A—deionised water/formic acid 0.1%; MP B—ACN/formic acid 0.1%; sample preparation—83 mg of 1-(3-benzoylphenyl)ethenone dissolved in 10 mL of methanol and filtered through a 0.45 PVDF filter; flow rate—68 mL/min; injection volume—1.2 mL; between-run equilibration—15,5 min of 32% MP B; UV detector set at 233 nm; column size—30.0 × 150 (id × length, mm); phase—Eclipse Plus C18, 5 µm; HPLC system—Agilent 1200 Prep System (Agilent Technologies, USA). The gradient elution programme is presented in Table 7.

### 3.4. IR

The IR spectrum of the material was registered in a disc of potassium bromide (≥99%, Merck, Germany; 1 mg of dried and ground in an agate mortar material in 300 mg of potassium bromide) in the region of 4000 to 400 cm^−1^ using a Nicolet iS5 (Thermo Fisher Scientific, Horsham, UK) infrared spectrometer.

### 3.5. NMR

The ^1^H and ^13^C NMR spectra of the material solution in deuterated chloroform (≥99.8% D atoms, Sigma-Aldrich, USA) were registered on a Bruker AVANCE III 400 MHz UltraShield Plus spectrometer (Bruker, Billerica, MA, USA) with operating frequencies of 400 MHz and 100 MHz, respectively.

### 3.6. Mass Spectrometry

Mass spectra were registered on an Agilent 1260 Infinity II liquid chromatograph equipped with a G7117A photodiode array detector, a G7115A spectrophotometric detector, and an Agilent 6470 mass spectrometric detector with ESI ionisation for positively charged ions.

## 4. Conclusions

The advancement of pharmaceutical chemistry and high-precision methods of pharmaceutical quality control require the use of high-quality RMs. The development of impure materials is a complex and challenging task. It is necessary to consider the intended use of the RM, the nature of impurity formation in MPs, as well as its individual physical and chemical properties. In this study, we have shown the possibility of using preparative chromatography to purify RM for regulatory requirements. We have also demonstrated the need to establish the authenticity of RMs using several independent methods of investigation based on different principles, such as mass spectrometry, IR, and NMR.

## Figures and Tables

**Table 1:** Purity of crude material 1-(3-benzoylphenyl)ethenone.

Peak Name	RT (min)	Area	Area (%)	Height
RTT 0.956	18.93	191,950	1.7437	76,780
Impurity A	19.80	9,957,204	90.4542	1,659,534
RTT 1.747	34.59	583,328	5.2991	145,832
RTT 1.766	34.96	275,520	2.5029	78,720

**Table 2:** Composition of the mobile phase at the time of elution of the components.

Peak Name	RT (min)	Mobile Phase Composition with Consideration of Elution Delay. (Mobile Phase B (%))
RTT 0.956	18.93	41
Impurity A	19.80	43
RTT 1.747	34.59	63
RTT 1.766	34.96	64

**Table 3:** Material purity of 1-(3-benzoylphenyl)ethenone after preparative HPLC.

Peak Name	RT (min)	Area	Area (%)	Height
RTT 0.956	18.9	16,648	0.1394	2081
Impurity A	19.8	11,926,584	99.8606	1,490,823

**Table 4:** Frequencies of the main absorption bands of the material 1-(3-benzoylphenyl)ethenone.

Absorption Maximum (nm)	Vibrational Mode	Attribution
3073–2918	ν C–H	–CH_3_, –CHAr
1684	ν C=O	15 C=O
1655	ν C=O	13 C=O
1595	σ C–C	–C–CAr
1427	σ_as_C–H	–CH_3_
1356	σ_s_C–H	
1304–1233	ν_as_C–CO–C	–C–CO–C
1175–1144	ν_s_C–CO–C	
820	ω C–H	–C–HAr
723–691	σ C–C	–C–CAr

**Table 5:** Description and assignment of ^1^H NMR signals of 1-(3-benzoylphenyl)ethanone material.

Chemical Shift (ppm)	Multiplicity	SSC J (Hz)	Intensity	Attribution
2.61	Singlet	-	3	16
7.46	Triplet	7.7	2	1, 2, 3, 8
7.57	Quartet	7.5	2	
7.76	Doublet	7.3	2	4, 6
7.94	Doublet	7.7	1	7, 9
8.14	Doublet	7.8	1	
8.33	Singlet	-	1	11

**Table 6:** Description and assignment of ^13^C NMR signals of 1-(3-benzoylphenyl)ethanone material.

Chemical Shift (ppm)	Attribution	Chemical Shift (ppm)	Attribution
26.6	16	132.8	7
128.4	1, 3	134.1	11
128.6	2, 8	136.9	5, 10, 12
129.5		137.1	
129.9	4, 6	138.0	
131.6	9	195.7	13, 15
		197.1	

**Table 7:** Gradient elution programme.

Time (min)	Mobile Phase B (%)
0 → 13	32 → 52
13	65
20	65

## Data Availability

The original contributions presented in this study are included in the article. Further inquiries can be directed to the corresponding author.
